# PTK-7 Expression in Gastric Cancer: A Prognostic Determinant

**DOI:** 10.4274/balkanmedj.galenos.2019.2019.8.12

**Published:** 2020-02-28

**Authors:** Melike Özçelik, Özlem Ercelep, Sevinç Keser, Nagehan Özdemir Barışık, Merve Başar, Hatice Odabaş, Abdilkerim Oyman, Selver Işık, Serhat Özçelik, Mehmet Aliustaoğlu

**Affiliations:** 1 Clinic of Medical Oncology, University of Health Sciences, İstanbul Ümraniye Training and Research Hospital, İstanbul, Turkey; 2 Clinic of Medical Oncology, Dr. Lütfi Kırdar Training and Research Hospital, İstanbul, Turkey; 3 Clinic of Pathology, Dr. Lütfi Kırdar Training and Research Hospital, İstanbul, Turkey; 4 Clinic of Endocrinology and Metabolism, Dr. Lütfi Kırdar Training and Research Hospital, İstanbul, Turkey

**Keywords:** Gastric cancer, prognostic factor, PTK-7

## Abstract

**Background:**

Protein tyrosine kinase-7, a regulatory protein in the Wnt signaling pathway, was highly overexpressed in various cancer types and assumed to be related to prognosis.

**Aims:**

The purpose of this study is to assess whether protein tyrosine kinase-7 expression status in curatively resected gastric carcinoma would independently identify patients with a high risk of recurrence and death.

**Study Design:**

Retrospective cohort study.

**Methods:**

We included patients who were at least 18 years of age and diagnosed with gastric cancer. The exclusion criterion was a metastatic disease at the time of diagnosis or operation. Data on clinicopathological prognostic determinants and clinical courses, including the date of disease relapse and survival status, were collected with the use of medical records. Surgically removed tumor tissue specimens were examined by two independent pathologists at the pathology department of our institution. Protein tyrosine kinase-7 expression status was assessed with immunohistochemical processing and stratified on a scale ranging from 0 to +3 according to the extent of stained tumor cells. It was then further categorized into two groups, one being + (positive), including +1, +2, and +3 scores, another was-(negative), including-and +/− scores.

**Results:**

A total of 114 patients were analyzed. Protein tyrosine kinase-7 expression was present in 66.7% of the surgical tumor specimens. There was no statistically significant difference in almost all relevant parameters between the protein tyrosine kinase-7 positive and negative groups. The estimated median survival in the protein tyrosine kinase-7 positive group was significantly better than the protein tyrosine kinase-7 negative group (60 vs 22 months, p<0.001). Disease-free survival was found to be 55 months in the protein tyrosine kinase-7 positive group, whereas it was 21 months in the negative group (p=0.015). In the multivariate analysis, along with negative protein tyrosine kinase-7 expression, poor performance status, and advanced stage were significantly associated with the risk of death (p<0.001 for each).

**Conclusion:**

Compared to patients with negative PTK-7 expression, patients with positive PTK-7 expression have better disease-free survival and overall survival rates. Efforts should be made to enhance this finding and translate it into clinical practice.

Gastric cancer is one of the leading causes of mortality worldwide (1). Unfortunately, despite today’s advanced technologies in medical and surgical treatments, survival rates are still very low for this type of cancer (2,3,4). Adjuvant chemo- and radiotherapy decrease relapse and mortality rates, but this benefit is coupled with side effects and resistance to treatment in early-stage gastric cancer (5,6). The outcome of gastric cancer is influenced by many factors, including disease extent, histologic type, the status of the resection margins, lymphovascular invasion, perineural invasion, tumor grade, and patient age (7,8,9). In addition to these well-established prognostic features, the development of prognostic biomarkers that match the molecular makeup of the tumor may lead to the development of more effective and safer therapeutic options by targeting the relevant mechanism responsible for tumor progression.

Protein tyrosine kinase-7 (PTK-7) is a protein that was first discovered in colon carcinoma cell lines. For this reason, it is also known as colon carcinoma kinase-4 (10). PTK-7 functions as a transmembrane cell surface glycoprotein that regulates the signal stimulation of downstream pathways. The major cellular pathway that PTK-7 involved in is the Wnt, both the non-canonical (also known as the wnt/planar cell polarity signaling) and the canonical (B-catenin dependent), signaling pathway, which regulates a variety of developmental processes, including adhesion, cell migration, cell polarity, proliferation, and actin cytoskeleton reorganization, along with apoptosis (11,12,13). The Wnt cascade also plays an important role in stem cell maintenance and differentiation (14,15).

Several tumor types, including breast cancer, non-small cell lung cancer, and sarcoma, exhibit constitutive activation of Wnt signaling through the autocrine secretion of ligands by a tumor cell. Recent evidence has shown that PTK-7 expression levels are increased in some tumor types and may be a potential target in cancer management (16,17,18,19). It has been argued that PTK-7 overexpression is associated with poor prognosis in most cancers. Some studies suggested an association with adverse clinicopathological features like aggressive histological subtype, lymph node metastasis, advanced tumor stage, lymphovascular invasion, and high grade (20,21,22,23). The poor outcome seen with PTK-7 overexpression provides an argument to discuss the potential benefits of adjuvant treatment after curative tumor resections. However, trials relevant to this topic in gastric cancer are limited and contradictory to the previous studies on other types of cancers. In the present study, we aimed to investigate the impact of PTK-7 expression on gastric cancer outcomes to determine its potential role as a therapeutic target.

## MATERIALS AND METHODS

### Study Design

This study was conducted in compliance with the ethical principles according to the Declaration of Helsinki, and it was approved by the local Institutional Review Board (July 29, 2016; protocol number: 2016/514/88/19). This retrospective follow-up study involved 114 patients who were at least 18 years of age, with curatively resected gastric carcinoma, diagnosed from 2006 through 2013 in the Department of Medical Oncology of our institution. Data on clinicopathological features, including age, gender, resection type, tumor location, histopathology, pT stage, tumor size, histological grade, resection margin, lymph node involvement, lymphovascular invasion, and perineural invasion were collected retrospectively. Clinical course, regarding the date of disease relapse and survival status, were also noted. Receipt of adjuvant treatment was determined based on the resection margin and dissection type along with the pathological stage. All patients with R1 resection or scheduled for adjuvant treatment with less than a D2 dissection received radiotherapy in addition to chemotherapy.

The seventh edition of the American Joint Committee on Cancer tumor, node, metastasis (TNM) classification was used for staging patients based on the information obtained from the pathological and radiological evaluation. The patients were eligible if they had histopathologically confirmed, curatively operated gastric cancer. The presence of metastatic or unresectable disease around the time of diagnosis or operation was the main exclusion criteria.

Post-operative surveillance after curative surgery was conducted through imaging procedures and serum tumor markers. Follow-up evaluations were done every three months in the initial two years after surgery, then every six months between the years three and five, and repeated at yearly intervals thereafter.

### PTK-7 evaluation

Surgically removed tumor tissue specimens were received and examined by two independent pathologists at the pathology department of our institution. PTK-7 expression status was assessed according to the manufacturer’s instructions of the kit. During immunohistochemical processing, three-micron slices were prepared from paraffin blocks and deparaffinized at 60 °C in an autoclave. Slides were labeled for the assay, and placed into the device (Leica Bond Max, serial no: M21284, Made in Melbourne Australia). Then, slides were placed into 5% hydrogen peroxide to block endogenous peroxide. Slides were incubated in monoclonal antibody CCK4 (ABCAM, LOT: GR170083-1, 1/500; England) and the Universal DAB kit, for 30 minutes. A postprimary antibody, polymer solution, and DAB mixtures (Leica Lot 11776) were applied for 10 minutes, respectively. Contrast staining was done with Mayer hematoxylin, and slides were closed with covering material.

**Figure 1:**
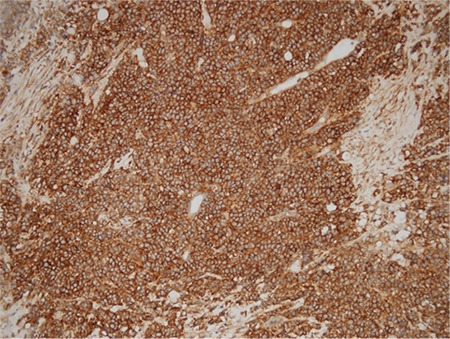
PTK-7, +++ /strong immunohistochemical staining (x200)

**Figure 2:**
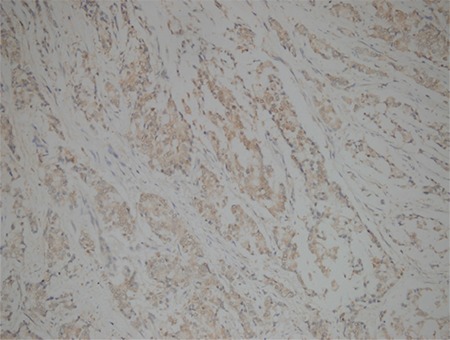
PTK-7, +/ weak immunohistochemical staining (x200)

We stratified PTK-7 expression, in line with the descriptive method in a previously performed study by Lin et al. (24), on a scale ranging from - to +++ according to the extent of stained tumor cells. Expression in >50% of tumor cells corresponds to +++ score, while 0% expression corresponds to a negative result. A ++ score was defined as expression between 20%-50%, + score as expression between 10%-20% and +/− score as expression in <10% of tumor cells (Figures 1 and 2). Estimated scores were then categorized into 2 groups, one being + (positive); including +, ++, and +++ scores, another was-(negative); including-and +/− scores.

### Statistical analysis

The characteristics of patients were evaluated with descriptive analysis. A Chi-squared test and Fisher’s exact test were used to compare the clinicopathological features between PTK-7 positive and negative subgroups. Overall survival (OS) was defined as the time from curative operation to death from any cause or to the last follow-up evaluation. Disease-free survival (DFS) was defined as the time between the curative operation and the earliest date of disease recurrence, death, or last follow-up. Patients who were lost to follow-up were censored at the last date they were known to be alive. Survival curves were estimated by the Kaplan-Meier method and compared across groups with the use of the log-rank test. We used a Cox proportional hazards model for the analysis of covariates as prognostic factors. A univariate analysis was performed initially. The following parameters were studied: PTK-7 expression, Eastern Cooperative Oncology Group (ECOG) performance status, TNM stage, T stage, gender, age, grade, tumor size, histopathology, resection type, resection margin, perineural invasion, vascular invasion, and lymph node status.

The independent impact of PTK-7 overexpression on the OS was evaluated using a multivariate Cox regression model adjusted for statistically significant prognostic factors. All variables were entered into the model and then removed by backward stepwise selection. The primary outcome was mortality. We calculated the effect size as 0.67 using a prediction of a 30% difference in mortality between the PTK-7 positive and negative groups according to a pilot test conducted before the study. A minimum of 35 patients per group was estimated to be required to provide a power of 80% at a significance level of α=0.05.

All statistical analyses were carried out using SPSS 17.0 version (IBM Corp., Armonk, NY, USA). A p-value below 0.05 was considered to indicate statistical significance.

## RESULTS

A total of 114 patients were included in this study. The baseline characteristics of the patients are detailed in Table 1. The median age was 62 years (range, 28-87 years). There were 45 females and 69 males. The primary tumor site was the antrum in 56.1% of patients. In all, 107 patients (93.9%) had adenocarcinoma, five patients (4.4%) had poorly cohesive carcinoma, signet ring cell subtype, and two patients (1.8%) had mucinous carcinoma. The resection margin status was R0 in 101 patients (88.6%) and R1 in 13 patients (11.4%). Fifty-three patients (46%) underwent D1 dissection and 61 patients (54%) underwent D2 dissection. All patients with R1 resection, or those scheduled for adjuvant treatment with less than a D2 dissection, received radiotherapy in addition to chemotherapy. Approximately 55% of patients were diagnosed with stage III cancer, whereas those with stage II and stage I accounted for 36.8% and 8.8% of the total, respectively. PTK-7 expression was present in 66.7% of the surgical tumor specimens. The association of PTK-7 expression with clinicopathological variables is summarized in Table 2. There was no statistically significant difference in almost all relevant parameters between the PTK-7 positive and negative groups. Only the resection type differed; compared with PTK-7 positive tumors, a significantly higher proportion of PTK-7 negative tumors underwent total gastrectomy (p=0.017).

In the PTK-7 positive and the PTK-7 negative groups, median follow-up times were 28 and 17 months, respectively. During follow-up, 43 patients (37.7%) had recurrent disease.

**Figure 3:**
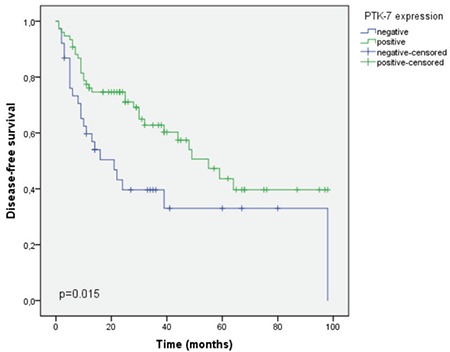
Improved disease-free survival in patients with PTK-7 positive tumors

**Figure 4:**
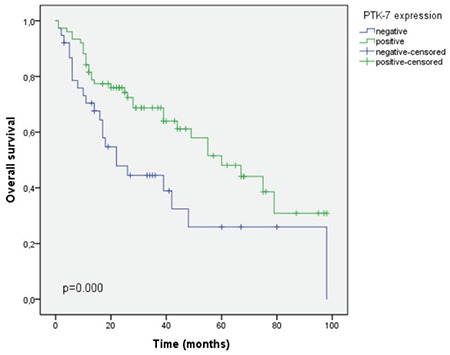
Improved overall survival in patients with PTK-7 positive tumors

The estimated median survival in the PTK-7 positive group was significantly better than the PTK-7 negative group (60 vs 22 months, p<0.001). Likewise, DFS was found to be 55 months in the PTK-7 positive group, whereas it was 21 months in the PTK-7 negative group (p=0.015). DFS and OS analyses are presented in Figures 3 and 4, respectively.

In the univariate analysis, PTK-7 expression status (p=0.016), ECOG performance status (p<0.001), TNM stage (p<0.001), T stage (p=0.015), vascular invasion (p=0.002), lymph node status (p=0.001) were found to be factors predictive of the OS duration (nagelkerke R2=0.422). However, a PTK-7 negative tumor status was independently associated with a worse OS (HR: 0.3; 95% CI: 0.1-0.5; p<0.001). In the multivariate analysis, along with negative PTK-7 expression, poor performance status, and advanced stage were significantly associated with the risk of death (p<0.001 for each). Univariate and multivariate analyses of predictors of the OS are shown in Table 3.

## DISCUSSION

Understanding the biologic processes that promote and sustain cancer is critical. Thus, the discovery of new targets will lead to the development of individualized management of patients. We sought to determine the potential impact of PTK-7 expression levels, on gastric cancer outcomes. Our data demonstrated significantly longer DFS and OS durations with PTK-7 positive tumor specimens of patients who underwent resection with curative intent of their gastric cancer.

PTK-7, a component of a complex signaling network of the wnt pathway that is considered to be dysregulated in various types of cancer, was found to be highly overexpressed in tumor specimens compared with normal tissue samples in most published data (16,22,23,24,25). However, little consistency was found across the studies regarding the role of PTK-7 in oncologic outcomes, with some suggesting a poor prognosis, whereas others, proposing a better outcome for expression positive tumors. The presence of PTK-7 expression portends a worse prognosis in many series. Jin et al. reported that the overexpression of PTK-7 was associated with poor DFS and OS in intrahepatic cholangiocarcinoma (22), and the expression was highly restricted to tumor samples rather than normal bile duct tissue. In another study examining PTK-7 expression in both primary breast tumors and their lymph node metastases, expression positivity in lymph node metastasis was significantly associated with shorter DFS (23).

Moreover, PTK-7 expression was correlated with the triple-negative phenotype, further supporting its adverse impact on breast cancer behavior. In a more recent study, Dong et al. (25) observed that PTK-7 expression was positively correlated with lymph node metastasis, TNM stage, and grade in oral tongue squamous cell carcinoma. Patients with high expression had a poor OS compared with negative and weak expression defined on a 0 to +3 scale (25). In a retrospective study involving 180 patients with prostate cancer, the authors revealed that elevated PTK-7 expression was significantly associated with lymph node metastases, seminal vesicle invasion, prostate cancer stage, the higher preoperative prostate-specific antigen, the higher Gleason score, angiolymphatic invasion, and biochemical recurrence (20). Lhoumeau et al. (21) confirmed the adverse prognostic effect of PTK-7 overexpression, finding out a significant association with reduced metastasis-free survival in non-metastatic patients with colorectal cancer. However, expression was not found to be associated with any specific clinical or pathological features, including age, tumor size, lymph node involvement, stage, lymphovascular invasion, colloid mucous component, and differentiation grade. In a very recent study, investigating the prognostic role of the PTK-7 in 85 patients diagnosed with cervical carcinoma, overexpression of PTK-7 was associated with malignant clinicopathological features, suggesting shorter progression-free survival (26).

Contrary to previous studies, however, in our study, the median OS for the PTK-7 positive tumors was 60 months compared with 22 months for PTK-7 negative cases. A recent trial, including 209 surgically resected colorectal cancer patients, has come to the same conclusion (27). The PTK-7 expression rate was found to be 68.3%, which closely resembled our findings of 66.7%. The authors reported that negative PTK-7 expression was associated with poor differentiation, lymph node metastasis, and advanced TNM stage. PTK-7 expression was correlated with longer survival time. As such, in a multivariate analysis, PTK-7 expression, along with vascular invasion, was found to be an independent variable of OS. We failed to demonstrate the correlation of PTK-7 expression with established clinicopathological determinants of prognosis. Our small patient population could be a possible explanation for this outcome. Lin et al. (24) performed a retrospective study in 201 patients diagnosed with stage I-IV gastric cancer. PTK-7 expression was noted in 56.7% of patients and was stronger in cancer tissue than in matched non-cancerous mucosa. PTK-7 was found to be correlated with well-differentiated tumors and was associated with improved DFS and OS. Stage, grade, lymphovascular invasion, hepatic metastasis, and radical resection were the other independent parameters of OS. They did not include performance status in the Cox regression analysis. In our study, we identified the ECOG PS as having prognostic relevance for OS time in addition to PTK-7 expression and stage. In the subgroup analysis of their trial, in stage IV patients, positive PTK-7 expression did not show favorable OS and DFS. We focused only on patients with non-metastatic tumors at diagnosis to avoid a heterogeneous population, which is a source of potential confounding bias. In this regard, we excluded tumors that were diagnosed as stage IV at the time of operation.

The prognostic significance of PTK-7 expression in many cancers has not been settled. One proposed explanation for this is the role of PTK-7 is still not well-understood and may differ in each tumor type, compounding the difficulty of interpreting data. Another is that the existing studies are limited by the performance of the different methods for defining expression. Using a 0-10 scale, 0%-100% scale, histological scoring method, and referring to the validation results of another study are the most frequently chosen methods for calculating staining intensity across studies. In our study, the descriptive method of PTK-7 expression was derived from the research conducted by Lin et al. (24). Despite considerable conflicting results in various types of cancer, in keeping with the previous study, we used the same calculation method and showed that the expression of PTK-7 appeared to predict longer survival time in gastric cancer patients.

Several studies suggest that the presence of PTK-7 may contribute to the development of clinical relapse. However, the data we have in most cancers are not fully reproducible in gastric cancer. PTK-7 serves as a modulator of better survival in gastric cancer. Additional oncogenic pathways are possibly involved in PTK-7 regulation in gastric cancer. The other possible explanation is that the enrolled patients may not be typical of the wider population as most studies suffer from the small number of included patients.

There are some limitations to this study. First, the study was retrospective and, therefore, subject to possible selection bias. Second, we could not address whether PTK-7 expression in tumor tissue was higher than in normal mucosa as it was shown in most previous studies. Last is the sample size was relatively modest.

In conclusion, the observation that PTK-7 status is independently associated with survival in gastric cancer patients supports the importance of it as a determinant of gastric tumor behavior. Current published data are promising, but the results still have not been translated into clinical practice. It would be useful to include the expression of PTK-7 in models predicting gastric cancer survival and to use the current clinicopathological system as a general guide in conjunction with the molecular stratification when treatment decisions are made.

## Figures and Tables

**Table 1:**
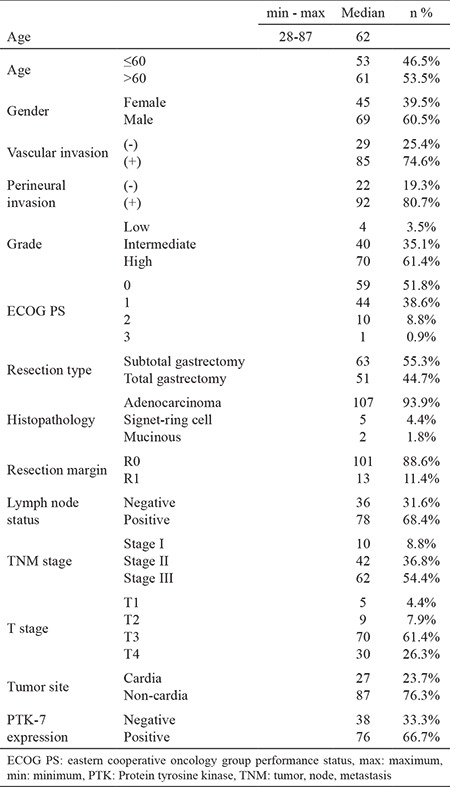
Baseline characteristics of patients

**Table 2:**
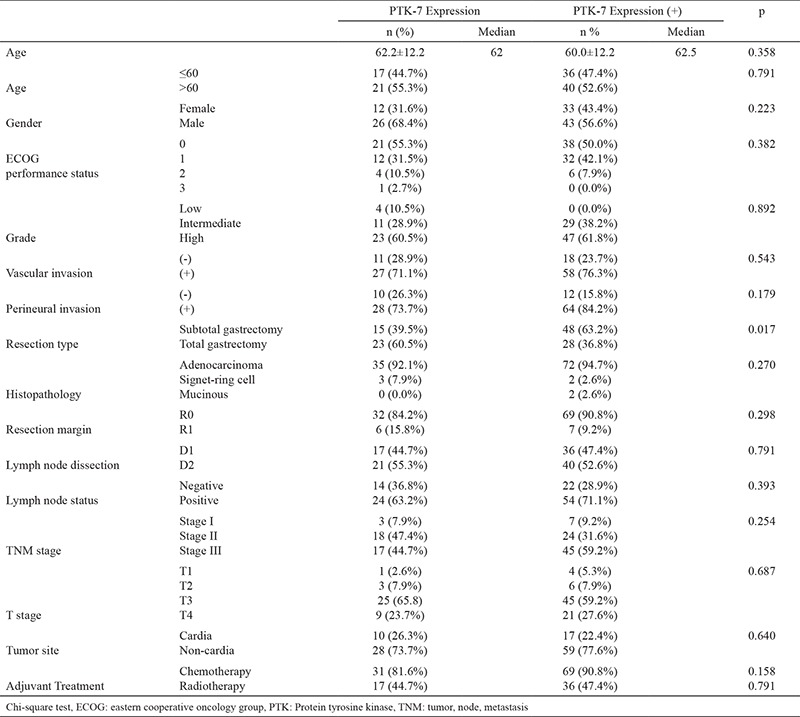
Association of PTK-7 expression with clinicopathological variables

**Table 3:**
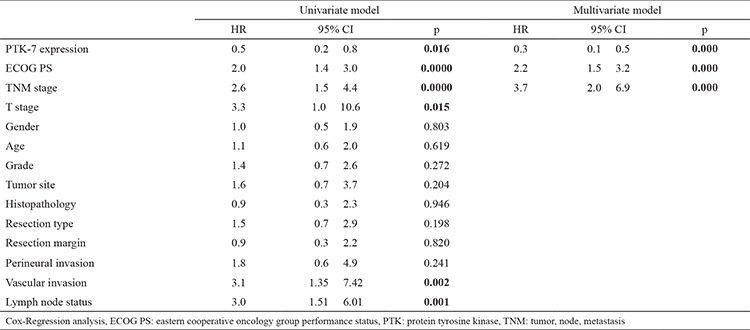
Univariate and multivariate analysis of predictors of the overall survival
